# Œsophageal Pouch

**Published:** 1898-04-16

**Authors:** 


### ŒSOPHAGEAL POUCH.

This condition, attention to which was lately drawn by Mr. Butlin, is probably not as uncommon as some people have supposed. A case is recorded in the *British Medical Journal* of a man, aged 45, who, when he was a boy, had swallowed a toy metal disc, but does not remember that he has had any serious trouble with his throat until 10 years ago, since when he has had difficulty in getting his food down, and after he has finished eating some food lodges in his throat, and is regurgitated. He has no pain, and has never brought up any blood. If he holds his head down after eating some of the food returns, and if it has lodged for an hour or two the regurgitated matter is frothy, but it never tastes sour. He can usually take solids, but sometimes at the beginning of a meal cannot swallow at all. After a meal overnight he can always bring up some of his food the next morning. By pressure on the right side of the neck above the sterno-clavicular joint he can force food up into the mouth, and such pressure causes air to escape with a squeaking noise. After drinking, which he does with effort, pressure on the sides of the neck causes escape of fluid and gas into the mouth. Since being advised to empty the pouch by pressure after each meal he has been much more comfortable.

